# Preventing female genital mutilation in high income countries: a systematic review of the evidence

**DOI:** 10.1186/s12978-019-0774-x

**Published:** 2019-07-22

**Authors:** Carolyne Njue, Jamlick Karumbi, Tammary Esho, Nesrin Varol, Angela Dawson

**Affiliations:** 1 The Australian Centre for Public and Population Health Research, Faculty of Public Health, University of Technology Sydney, PO Box 123, Broadway, Sydney, NSW 2007 Australia; 2 Ministry of Health, PO Box 30016-00100, Nairobi, Kenya; 3 Community and Public Health, Technical University of Kenya, PO Box 52428-00202, Nairobi, Kenya; 4 Sydney Medical School, The University of Sydney, 135 Macquarie St, Sydney, NSW 2000 Australia

**Keywords:** Female genital mutilation, Migrants, Health prevention, Systematic review, High-income countries

## Abstract

**Background:**

Female genital mutilation (FGM) is prevalent in communities of migration. Given the harmful effects of the practice and its illegal status in many countries, there have been concerted primary, secondary and tertiary prevention efforts to protect girls from FGM. However, there is paucity of evidence concerning useful strategies and approaches to prevent FGM and improve the health and social outcomes of affected women and girls.

**Methods:**

We analysed peer-reviewed and grey literature to extract the evidence for FGM prevention interventions from a public health perspective in high income countries by a systematic search of bibliographic databases and websites using appropriate keywords. Identified publications were screened against selection criteria, following the PRISMA guidelines. We examined the characteristics of prevention interventions, including their programmatic approaches and strategies, target audiences and evaluation findings using an apriori template.

**Findings:**

Eleven documents included in this review described primary and secondary prevention activities. High income countries have given attention to legislative action, bureaucratic interventions to address social injustice and protect those at risk of FGM, alongside prevention activities that favour health persuasion, foster engagement with the local community through outreach and the involvement of community champions, healthcare professional training and capacity strengthening. Study types are largely process evaluations that include measures of short-term outcomes (pre- and post-changes in attitude, knowledge and confidence or audits of practices). There is a dearth of evaluative research focused on empowerment-oriented preventative activities that involve individual women and girls who are affected by FGM. Beattie’s framework provides a useful way of articulating negotiated and authoritative prevention actions required to address FGM at national and local levels.

**Conclusion:**

FGM is a complex and deeply rooted sociocultural issue that requires a multifaceted response that encompasses socio-economic, physical and environmental factors, education and learning, health services and facilities, and community mobilisation activities. Investment in the rigorous longitudinal evaluation of FGM health prevention efforts are needed to provide strong evidence of impact to guide future decision making. A national evidence-based framework would bring logic, clarity, comprehension, evidence and economically more effective response for current and future prevention interventions addressing FGM in high income countries.

## Plain English summary

Female Genital Mutilation (FGM) is a cultural practice that is associated with poor health outcomes. The migration of women and girls with FGM to countries such as the United Kingdom, the United States of America and Australia, where FGM is not traditionally practiced, has led to the development of programs to prevent FGM and care for affected women and girls. The purpose of this study is to identify and review existing literature to find out what strategies and approaches are useful to prevent FGM and improve the health of affected women and girls. The majority of the documents we included in this review described laws developed to prevent FGM and safeguard girls and women, as well as media campaigns and health education activities for health professionals and community members. All the evaluations measured short term outcomes such as improvements in knowledge and we did not find any evidence of long term changes. There is a need to carry out well designed evaluations to understand how interventions can change behaviour. This evidence can then be used to inform national plans to prevent FGM.

## Background

WHO defines female genital mutilation (FGM) as “all procedures that involve the partial or total removal of the external female genitalia, or other injury to the female genital organs for nonmedical reasons” [1]. WHO classifies FGM into four categories with type III (infibulation) being the most severe form [1]. This is a more detailed description of practices than the one offered by UNICEF [2]. FGM, also known as female genital cutting or female circumcision, has serious health consequences that may lead to death from haemorrhage and/or infection, urinary and genital tract infections, gynaecological, obstetric, sexual and psycho-social complications or death from haemorrhage [3–5]. While the prevalence of FGM is decreasing and varies across countries [6] UNICEF has estimated that more than 200 million of girls and women have undergone Female Genital Mutilation (FGM) globally and three million girls may be at risk of undergoing FGM every year [7]. FGM occurs in more than 40 countries throughout the world. It is practiced by communities in 28 African countries, communities in the southern parts of the Arabian Peninsula and along the Persian Gulf and in communities in India, Indonesia and Malaysia [8, 9].

Difficult economic conditions and conflict are among the factors that have resulted in increasing migration of people from FGM prevalent nations to high income countries (HIC) where FGM is not traditionally practiced such as the United States of America (USA), United Kingdom, Australia, New Zealand, and across Europe [9]. Many of these women and girls would be at risk of undergoing FGM in their countries of origin. Some migrants have continued the practice in their countries of migration, with the aim of maintaining their culture and identity [10, 11]. However, there is evidence that migration to countries where FGM is not prevalent has a positive influence on the abandonment of this practice [12–16]. Some of the reasons cited for this change include improved knowledge of the health consequences and weakening of social pressure to undertake FGM that is associated with changing perceptions regarding the benefits of the practice for marriageability, religious observance and social acceptance. Moreover, those affected by FGM may be stigmatised in their new country of migration [17]. FGM is a prosecutable offence in most HIC. Whilst this may dissuade communities from undertaking FGM, it may also encourage families to conduct FGM in secret or take their children overseas to be circumcised.

The exact number of women and girls living with FGM in HIC is largely unknown due to the sensitive nature of the issue and the lack of routinely collected data. However, it is estimated that almost half a million women living in Europe have been subjected to the practice [18]. In the United Kingdom (UK) and USA, based on 2015 estimates, there are about 137,000 women and girls who have undergone FGM and 507,000 who are at risk of FGM [19, 20]. Health professionals in HIC are often not aware of FGM or have knowledge and skills to adequately care for affected women or protect children at risk. FGM therefore presents a challenge to the health system in HIC [21, 22].

Given the harmful effects of this practice and its illegal status in many countries, there have been concerted efforts to prevent FGM in HIC through advocacy and other prevention activities including education, information and public communication campaigns for affected women and girls. Mandatory reporting protocols have been developed in some countries, such as the United Kingdom (UK), to protect women and girls at risk. In addition, there are police protocols and legal means to prosecute those suspected of carrying out the practice [23] However, there is not enough evidence concerning effective strategies and approaches to prevent FGM and improve the health and social outcomes for affected women and girls. Four recent reviews have largely focused on interventions from African countries [24–27]. We therefore undertook a systematic review to identify the evidence for FGM prevention interventions from a public health perspective in HIC. Specifically, we examined the characteristics of interventions, including their programmatic approaches and strategies, target audiences and evaluation findings. The review aims to contribute to improving the knowledge base to inform the design and evaluation of FGM health interventions to prevent the practice and optimize health outcomes for girls and women with FGM.

## Methods

Due to the short-term nature of the largely process evaluations we identified that used a range of methodologies, we were not able to pool data to establish the overall effect of the interventions on prevalence. This determined the use of a qualitative content analysis rather than a quantitative meta-analysis to establish effectiveness. We undertook a systematic review and content analysis using an apriori framework to analyse the characteristics and underpinning approaches of interventions to prevent FGM in HIC and their outcomes. This review was registered with the International Prospective Register of Systematic Reviews as PROSPERO CRD42018092299.

For the purposes of this review, the term HIC is based on the World Bank definition, which divides member countries into different income groups. A HIC is as a country with a higher gross national income per capita of $12,376 USD [28]. We defined an ‘FGM intervention’ as any form of action or deliberate process to interfere with, modify or change individuals’ or groups of people’s knowledge, attitudes or behaviours to prevent FGM and thereby reduce the prevalence and improve the health outcomes of girls and women affected by FGM. We used Tannahill’s health promotion approach [29] to define types of interventions for inclusion and as such, clinical interventions were excluded. However, education programs to support affected women who may have undergone de-infibulation for example, were part of this review as was legislation to protect girls and community awareness to prevent FGM. We selected Tannahill’s model of health promotion where prevention is a key pillar [29] because it acknowledges, as other authors have noted, that prevention cannot be achieved without health promotion [30]. We aimed to understand the different strategies that are used to prevent FGM that reveal power dynamics and possible ethical dilemmas. This understanding informed the use of Bettie’s model [31] to further explore these strategies that recognises that prevention and health promotion are conceptually linked.

To achieve a comprehensive systematic search and hence retrieve all contemporary empirical evidence, we assessed all types of literature, ranging from grey literature reports to peer-reviewed literature concerned with the evaluation of interventions aimed at preventing and/or providing education for girls and women with FGM in HIC. The search for evidence was limited to what was available online and snowballing from bibliographies of retrieved articles.

A set of keywords used in the search were ‘female genital mutilation/cutting’, ‘female genital mutilation,’ ‘female genital cutting,’ ‘female circumcision,’ ‘clitoridectomy,’ ‘excision,’ ‘infibulation,’ ‘sunna,’ ‘FGM intervention,’ ‘FGM/FGC’, ‘FGM or FGC program, ‘high income country,’ developed country,’ and ‘developed regions’. Peer reviewed literature was identified using PubMed and CINHAL (EBSCO), EMBASE and Web of Science. We further searched institutional websites and databases of organizations involved in FGM activities, to identify reports and any possible “grey literature.” We conducted general searches using Google and Google Scholar to ensure no literature was missed. In addition, we cross-referenced articles, which were relevant and scanned through their bibliography to identify additional materials.

**Fig. 1:**
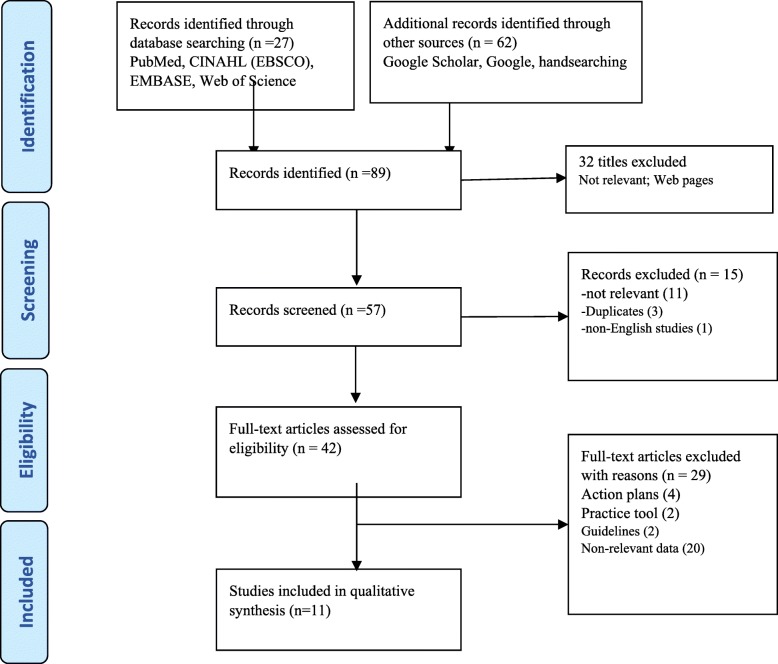
Flow Chart

The first author (CN) identified studies for relevance based on the title and abstract and the third author (TE) repeated the process to ensure no relevant studies were excluded. We included studies that focus on assessments or evaluations of FGM interventions published since 2000 as being eligible. We used a pre-established eligibility and inclusion and exclusion criteria to guide the screening and selection process. The inclusion criteria used were the following: (i) countries described were from a HIC setting as defined above; (ii) were conducted in recent years (January 2000–March 2018); (iii) the studies focused on assessing the impact of FGM interventions; and (iv) were in English. There was no restriction; all study types, designs and methodologies, those appearing in peer review journal or in grey literature from a recognised institution and/or government, or a PhD thesis, were all included so long as there was a clear methodology to enable an assessment of quality. Studies were excluded if they were from low or middle-income countries (LMIC), if they assessed consequences of FGM, described national action plans, were comprised of theoretical notes and recommendations/guidelines, or if they were not in English. Our search located 87 study reports that were eligible for review as shown in the PRISMA flow chart [32] (Fig. 1).

Following the screening, 42 study reports were identified as eligible for full text review. These were retrieved and assessed by the second author (JK) and counterchecked by first author (CN). The authors (JK and CN) independently appraised the studies for quality of reporting, this included an assessment of its quality, size and consistency of the body of evidence (See Tables 1 and 2). The study type, design, and methodology determined the type of quality assessment tool used and data were recorded on Microsoft Excel software.

**Table 1:** Rating of studies reviewed, using principles of quality outlined in the CASP tool

Reference	Study type (code)	Clear statement of the aims of the research	Appropriateness of methodology	Appropriateness of research design	Appropriate recruitment strategy	Appropriate data collection	Relationship between researcher and participants been adequately considered	Ethical issues been taken into consideration	Rigorous data analysis	Clear statement of findings	Findings in context (Contribution to body of knowledge)	Quality of study
		1 = Yes, 0 = Can’t Tell, − 1 = No	1 = Yes, 0 = Can’t Tell, − 1 = No	1 = Yes, 0 = Can’t Tell, − 1 = No	1 = Yes, 0 = Can’t Tell, − 1 = No	1 = Yes, 0 = Can’t Tell, − 1 = No	1 = Yes, 0 = Can’t Tell, − 1 = No	1 = Yes, 0 = Can’t Tell, − 1 = No	1 = Yes, 0 = Can’t Tell, − 1 = No	1 = Yes, 0 = Can’t Tell, − 1 = No	1 = Yes, 0 = Can’t Tell, − 1 = No	
Johansen et al., 2018 [33]	P	1	1		0	1	1	0	0	1	1	Moderate

**Table 2:** Rating of studies reviewed, using principles of quality outlined in the AACODS checklist

Reference	Country	Authority			Accuracy^a^					Coverage	Objectivity$		Date		Significance				Quality of study
		Reputable Individual author?	Reputable Organisation or group?	Detailed reference list	clearly stated aim	stated methodology	Peer-reviewed?	Data collection explicit and appropriate for the research?	Edited by a reputable authority?	Are any limits clearly stated? E.g. Particular population or group	The author’s standpoint clear?	Does the work seem to be balanced in presentation?	Clearly stated date related to content?	Key contemporary material been included?	Item meaningful? (this incorporates feasibility, utility and relevance)	Add context?	Strengthen or refute a current position?	Does it have impact? (in the sense of influencing the work or behaviour of others)	
		1 = Yes, 0 = Can’t Tell, −1 = No	1 = Yes, 0 = Can’t Tell, − 1 = No	1 = Yes, 0 = Can’t Tell, − 1 = No	1 = Yes, 0 = Can’t Tell, − 1 = No	1 = Yes, 0 = Can’t Tell, − 1 = No	1 = Yes, 0 = Can’t Tell, − 1 = No	1 = Yes, 0 = Can’t Tell, − 1 = No	1 = Yes, 0 = Can’t Tell, − 1 = No	1 = Yes, 0 = Can’t Tell, − 1 = No	1 = Yes, 0 = Can’t Tell, − 1 = No	1 = Yes, 0 = Can’t Tell, − 1 = No	1 = Yes, 0 = Can’t Tell, − 1 = No	1 = Yes, 0 = Can’t Tell, − 1 = No	1 = Yes, 0 = Can’t Tell, − 1 = No	1 = Yes, 0 = Can’t Tell, − 1 = No	1 = Yes, 0 = Can’t Tell, − 1 = No	1 = Yes, 0 = Can’t Tell, − 1 = No	
Brown & Porter, 2016. [34]	UK	1	1	−1	1	1	0	1	0	0	1	1	1	0	1	1	1	1	Moderate
Brown & Hemmings, 2013 [35]	UK	1	1	−1	1	1	0	1	0	0	1	1	1	0	1	1	1	1	Moderate
EIGE, 2013 [36]	EU and Croatia	0	1	1	1	1	0	1	1	1	1	1	1	1	1	1	1	1	High
Leye & Alexia, 2009 [37]	EU countries	0	1	0	1	1	0	1	0	1	1	1	1	0	1	1	1	1	High
Leye & Deblonde, 2004 [38]	EU countries	0	1	1	1	1	0	1	0	1	1	1	1	1	1	1	1	0	High
McCracken, Fitzsimons et al., 2017 [39]	UK	0	1	1	1	1	0	1	0	1	1	1	1	0	1	1	1	1	High
Mohamed, Schickler et al., 2014 [40]	UK	0	1	1	1	1	−1	1	0	1	1	0	1	1	1	0	1	1	Moderate
Scott & Jerse, 2011 [41]	Australia	0	1	0	1	1	0	1	0	1	1	0	1	0	1	1	1	1	Moderate
Simret, D., 2014 [42]	Canada	0	1	1	1	1	−1	1	0	1	1	1	1	1	1	1	1	0	High
NHS England 2016 [43]	England	0	1	−1	1	1	0	0	1	−1	1	0	1	1	1	1	1	1	Moderate

^a^This domain was rated No, where there was lack of peer review and details about the editors. $ A majority of our evidence being from programmes were not peer reviewed and hence scored no. Ratings: 0–6 being Low quality; 7–11 being moderate quality; 12–17 represents high quality

Of the 42 documents, 11 met the inclusion criteria (See Table 3). Evidence from ten grey literature was assessed for quality using Authority, Accuracy, Coverage, Objectivity, Date, and Significance (AACODS) Checklist [45]. We used Critical Appraisal Skills Programme (CASP) checklists to appraise one study published in peer-reviewed journals [46]. Data was extracted from the findings’ sections of the included papers (ad-verbatim and marked in quotation marks) in the form of sentences, phrases, or text units dealing the effectiveness of an intervention to identify patterns according to each intervention. Data extraction was undertaken by the authors (CN and JK) using a predesigned table. This was counterchecked by fourth (NV) and fifth (AD) authors.

**Table 3:** Characteristics of included studies and type of intervention, associated strategies and findings

Reference/ context	Aim	Methodology / Sample	Prevention activities examined	Findings: intervention outcomes
Primary prevention				
Johansen et al., 2018. [33] Australia, Austria, Belgium, Finland, France, Germany, Greece, Ireland, Italy, Netherlands, Norway, Portugal, Saudi Arabia, Slovakia, Spain, Sweden, Switzerland, UK, USA	To examine Countries national policies on FGM and health sector involvement in the management of FGM	Mixed methods: desktop review, survey with 21 respondents in 19 countries	•Training of health care professionals (HCP), involving HCP in the preventive work •Legal regulations on HCP duties to avert and report as well as to perform FGM •Availability of healthcare services (de-infibulation, clitoral reconstruction, sexual and psychological counselling	•4 of the 19 countries (France, Ireland, the Netherlands and Norway) had policies on FGM that were implemented, funded, coordinated and with systems of monitoring and evaluation in place; •15 countries had national policy on FGM, (exception Austria, Greece); • 12 countries had assigned coordination bodies (exception Australia, Austria, Greece, Sweden, and USA); 13 have partially or fully implemented the plans (exception: Austria, Greece, Spain, Sweden,); HCPs are legally prohibited from performing FGM on minors in •Psychological and sexual counselling were available predominantly in 13 countries
Leye & Deblonde, 2004 [38], Austria, Belgium, Denmark, Finland, France, Germany, Greece, Ireland, Italy, Luxemburg, Portugal, Spain, Sweden, Netherlands, UK	To examine FGM legislation specifically: Criminal laws, Child/Minor Protection laws, Specific FGM legislation, General Criminal Law, Professional secrecy law.	Mixed methods: -content analysis of legal documents, semi-structured interview with key informants in 5-member states (Belgium, France, Spain, Sweden, UK) (number of participants in each country not stated)	• Legal provisions pertaining to FGM	• 5 countries have child protection guidelines or protocols that guide those who are confronted with a girl at risk of FGM, except in Belgium • Cases are reported, and investigations have been initiated, although the number of cases brought to criminal court is limited (in France and Spain) • Issues in relation to type IV and piercing and cosmetic surgery where the latter has not been taken into consideration by legislators hence ambiguity. • Lack of knowledge of the law among health professionals, authorities and police. • Attitudes of these people may obstruct implementation i.e. fear of being labelled as racist • Workshops for professionals raised awareness of FGM
Leye & Alexia, 2009 [44], Belgium, France, Spain, Sweden, United Kingdom	To examine the implementation of criminal and child protection laws on FGM in 5 EU member states and associated activities	Mixed methods: content analysis of documents, survey questionnaire with key informants, in-depth interviews with participants from 5 countries, (number not stated).	• FGM related laws • Awareness raising and education to improve knowledge and compliance with the law including capacity building workshops for professionals from various sectors (targeted training and information campaigns about FGM issues, legislation, child protection procedures)	• Legislation alone is not enough (Barriers - Case identification and Collection of sufficient evidence to mount a prosecution) • No evidence was found to state that specific criminal law provisions are necessary to guarantee the punishment of FGM, or that they are more successful in their implementation than general criminal law provisions. • Only France and Italy have had prosecutions
Mohamed, Schickler et al., 2014 [40], UK	To report on participatory workshops on FGM to increase knowledge and awareness of FGM	Qualitative. 36 grandmothers, 52 young mothers; 38 men were interviewed using a structured questionnaire	• 5 local women trained as community champions to conduct 10 participatory workshops on FGM • The champions held one-to-one sessions with women, home visits were also made to speak with families about FGM • Visits were made to mosques to educate men and to schools	• At the end of the workshops 25% of participants stated that FGM was required by religion compared with 40% before the workshops began. • At the end of the workshops 79% of participants agreed that FGM should be stopped compared with 62% at the start. • Stories of women informed the development of materials and scenarios for discussion • Principles developed for action
Scott & Jerse, 2011 [41], New South Wales, Australia	To evaluate the state wide FGM multidisciplinary model of service delivery.	Mixed methods: document, consultations with 72 stakeholder informants and on-line survey of 63 respondents.	• Continuing professional development for health and other professionals; • Education and community development with affected communities involving community workers; • Resource and information development and dissemination • Advocacy to prevention FGM including media campaigns on ethnic radio run over 8 weeks • Collaborative work with NSW Police Child Protection Unit	• Build strong links with the community • increased awareness, knowledge and empowerment of affected community members and increased understanding in a diversity of professional health and other community service providers.
Simret, 2014 [42], Winnipeg, Canada	To evaluate a community-based education program, 10 weeks of educational, health and sociocultural support sessions on FGM	Qualitative: focus groups, questionnaires and observations with 187 immigrants and refugee participants in 3 African national communities; 24 service providers & other professionals. Examination of staff logs, and documentation review.	• Manual of materials designed for services to use with migrant and refugee women •Networking with key community organisations • Workshops for health and social service professionals on cultural competence	• Increased knowledge of mainly “high” to “very high” of women on FGM issues; 88% “strongly agreed” that workshops were suited to their needs; healthcare professionals showed increase in knowledge; most “strongly agreed” that content & process of training were appropriate • Having community co-facilitators helps ease the communications and acceptability of the intervention
Secondary prevention				
NHS 2016 [41], Clinical commissioning groups (CCGs) in the London Region, UK	To conduct a performance assessment of all CCG’s safeguarding governance, arrangements and processes.	A formal assurance review was carried out quarterly in line with the published framework and technical guidance. Eleven audits, the Safeguarding Adults Board Self-Assessment Audit, Safeguarding Workforce Gap analysis and baseline review, serious untoward incidents and section 2 audits and the safeguarding adults review and SCR (Single Central Record) tracker for adults and children	• Clear policy for adult and children safeguarding that includes FGM • Service engagement around FGM	• 37% of CCGs were judged as needing to make improvements to put in place clear children and adult policies that make sufficient references to prevent FGM • Most CCG’s were assessed as outstanding were engaging with FGM policy, 21% (*n* = 7) were given this score.
Multi-prevention				
Brown & Hemmings, 2013 [35], England & Wales, UK Liverpool, Leicester, Cardiff, Middlesbrough, Manchester, Birmingham, Bristol, Bolton, & 6 projects in London	To evaluate community based preventive work to safeguard children from FGM	Qualitative: 15 stakeholder interviews, interviews with community members using PEER ethnographic evaluation, rapid policy mapping and review of self-reported M&E	*Primary* • Community engagement and awareness raising incorporating FGM messages in wider sexual health messages, provision of safe spaces for discussion on FGM, using community champions, and use of drama and visual arts. *Secondary* • Mainstreaming FGM under violence against women and girls /safeguarding strategies involving multiple agencies, clinical & community champions and community awareness raising	• Indication of change in community views. •Community engagement improved rejection of FGM and has built networks of groups working together • Younger women have been empowered to speak out and make decisions • Some religious leaders have dismissed the perceived religious basis for certain forms of FGM *Secondary* •Mainstreaming: resources are committed To FGM, sustainability of efforts is high
Brown & Porter, 2016 [34], England and Wales UK	To evaluate the support provided to community-based organisations to carry out FGM prevention work	Qualitative: PEER interviews with 51 participants	*Primary* • Community education to promote a rights-based approach to tackling FGM • Awareness raising of FGM amongst affected communities, policy-makers, statutory agencies, and the public • Networking of groups and policy-makers to contribute to wider campaign to end FGM • Building skills and capacity of affected communities *Secondary* • Safeguarding practices e.g. child protection, legal action •Developing materials- culturally appropriate means of talking about FGM as a part of child protection • Promoting access to services for girls and women at risk through strengthening links between groups and statutory agencies	• Groups where there was a visible shift towards speaking out against FGM included parents, grandparents and young women who either had undergone or were at risk of FGM. • Respondents felt that changes in attitudes, awareness, levels of information and opposition were closely linked to the work of the national-level anti-FGM campaign and in particular the community-level work of the ten projects • Discussions about FGM in many areas, which many reported has changed the status of FGM, with it no longer being viewed as a ‘taboo’ subject. • Law was seen as a powerful and effective part of ending FGM
EIGE, 2013, All EU countries and Croatia [36]	Analysis of FGM policy and legal frameworks of FGM in EU-27 countries and Croatia and to understand past and present good practices in relation to prevention, protection, prosecution, provision of services and partnerships.	Qualitative: desk review of laws. In depth interviews with at least 6 people in 9 countries and five in-depth interviews at European /international level.	*Primary* • FGM related law • Awareness raising activities to improve knowledge and compliance with the law including awareness raising aimed at the general public •Development of training material *Secondary* •Training professionals on child protection	• Currently, there are nine EU Member States which have specific criminal law provisions on FGM. • No EU Member State has a specific provision on international protection and FGM in its national legislation. • Sweden, the Netherlands and Italy have prioritised funding for prevention activities, but the majority of Member States have not. Prevention activities lack baseline data and are poorly evaluated *Secondary* • Training on child protection in relation to FGM appears random and does not seem to be conducted on a continuous, structured and nationwide basis. • In many Member States, health professionals cannot break their code of silence when the crime of FGM has already been performed, because FGM is not generally considered as a type of repetitive, recurrent child abuse
McCracken, Fitzsimons et al., 2017 [39] 3 local authority areas within London, UK	To evaluate a pilot FGM early intervention model involving clinics at local hospital midwifery services.	Qualitative: In-depth one-to-one semi-structured interviews with 24 professionals & 6 women & 1 FGD with 6 women. Structured observation of 5 stakeholder and community events.	*Primary* • Community advocates raise awareness at local events and identify women’s needs • Support & information provided to men; local and religious leaders • Engagement with students at schools • Local media used to raise awareness *Secondary* • Training and development of assessment tools and protocols for health and education professionals and social workers for safeguarding and referral. • Community advocates accompany women to the clinic *Tertiary* • Education provided to women with FGM by midwives, social workers and therapists at clinics	• At least 235 women with FGM were seen in clinics in London; study showed clinics offered referral pathways & education; services, confirmed physical & mental health problems in women with FGM • Holistic service was reportedly provided: mental health services; advice on effective safeguarding approaches; support to access wider services and benefits; links to community-based classes and activities; emotional and practical support. • Health and social care professionals, therapists and community advocates reported strong working relationships and effective service protocols.

We first categorized the FGM interventions studies according to different levels of prevention, namely: primary, secondary and tertiary, as per the public health approach [47] and examined the outcomes of each initiative. In addition to the levels of prevention and the associated outcomes, we then undertook a content analysis informed by Beattie’s model of health promotion to understand dimensions of power and the socio-cultural context of the interventions. Drawing on this model, we examined the mode and focus of interventions specifically how negotiated action was achieved or how authoritative interventions were successful at changing individual or group behaviour [31]. We mapped this across the four quadrants of the model.

## Results

A total of 11 study reports are included in this review produced across multiple agencies involved in delivering FGM programs in HIC. One study [33] discusses FGM programs across 19 HIC. Five reports discuss FGM programs in the UK [34, 35, 39, 40, 44]. Three reports outline work undertaken in EU member states [37, 38, 48] and two documents describe FGM efforts in Canada [42] {Daniel, 2014 #229} and Australia [41] {Scott, 2011 #220}. The 11 studies used qualitative and quantitative methods. Six studies were judged to be of high quality (five of these were based on the AACODs checklist) [37–39, 42, 48]. Five studies were judged to be of moderate quality (three of these were based on the AACODs checklist) [35, 40, 41] and one was based on the CASP checklist for qualitative studies [33] {Abreu, 2015 #222}. One study was judged to be of low quality based on the AACODs checklist [44]. The activities described in the 11 reports were classified according to their primary, secondary or tertiary prevention focus (Summary Table 2).

### Interventions focused on the primary prevention of FGM

All documents included in the review described primary prevention activities that sought to increase individual professional and community awareness and understanding of FGM as an infringement on human rights, as well as the adverse health outcomes associated with the practice and the law as it pertained to FGM. Studies also provided insight into the current legal status of FGM in a number of European countries and issues relating to the implementation of the law.

Mohammed and colleagues (2014) describe participatory peer-to-peer educational workshops in the UK that involved the training of local Somali women as community champions in Tower Hamlets, London [40]. These workshops covered a wide range of FGM related topics, including health and well-being, FGM legislation, and stories from women who had experienced FGM. A before and after workshop assessment to assess knowledge and attitudes was conducted that showed increased changes in knowledge acquisition, attitude, beliefs and intention relating to FGM. Additionally, one-on-one outreach sessions that involved 21 visits by either a project co-ordinator or the community champions to households to speak to families and to provide on-going support to the households allowed women to speak in confidence about FGM issues.

Two studies [41, 42] conducted in Canada and Australia report on the outcomes of broad public education campaigns that focused on improving community members and health professionals awareness of human rights issues and knowledge of FGM prevention. Daniel and colleagues (2011) describe a 10 weeks program of educational, health and sociocultural support sessions to discuss, compare and share stories targeting newly arrived African migrant and refugee women in Winnipeg, Canada. Moreover, a manual of materials for services in relation to sexual and reproductive health and FGM information and prevention was developed. They reported that many of the women felt that by virtue of living in a new culture, the decision not to circumcise daughters was easier [42]. Scott and Jerse (2011) reported that educational interventions in Australia may have some effect in changing attitudes and knowledge on FGM of migrant communities to abandon the practice, especially when community champions and bilingual community workers from the affected communities are actively engaged [41]. Similarly, Brown and colleagues in their UK studies showed that when the community is engaged in the design of outreach interventions for girls, women and men, awareness of FGM can be enhanced, enabling participants to talk freely about what had previously been considered a taboo topic [34, 35]. Community outreach from dedicated FGM clinical services in the UK were also found to be useful in raising awareness [44, 39].

The European Institute for Gender Equality (EIGE) report [48] notes that Sweden, the Netherlands and Italy have prioritised funding for prevention activities, but the majority of European Union Member States have not and that prevention activities lack baseline data and are poorly evaluated.

Four of the studies [37, 38, 44, 48] provide some insight into European laws to prevent FGM and associated activities to educate communities about the laws and support health and other professionals to implement them. The EIGE report states that in 2013 nine of the 27 EU Member States had specific criminal law provisions on FGM. However, no EU Member State had a specific provision on international protection and FGM in its national legislation [48]. According to Leye and Deblonde (2004), national legislation is a useful first step towards preventing FGM as it encourages institutions to take subsequent measures to prevent FGM but alone is not sufficient to stop FGM [38]. There is no evidence that the implementation of a specific law is more successful than general criminal laws to prevent FGM. Specific laws include child protection laws that maybe be applied in cases of FGM and special provisions with regards to professional secrecy and disclosure, which may be applied to cases of performed or planned FGM. General criminal laws including articles of the penal code may be applied to FGM-related cases.

Despite the development of laws, three studies included in the review note that it was very challenging to enforce these laws. France and Italy were cited as the only countries to have had prosecutions and even then, they involved long protracted processes [37]. One of the two main barriers for the implementation of legislation is the identification of cases, which is principally obstructed by the lack of knowledge of FGM among professionals. The second important barrier is the complexity of finding sufficient evidence to bring a case to court. The authors recommend that to be effective, different sectors and the relevant professionals need to be properly trained and involved in this process to ensure the harmonized implementation of criminal and child protection laws. The EIGE and Johansen et al. studies indicate that most EU countries have laws to prosecute adults involved in procuring FGM for girls outside of a country’s borders, although its implementation is not uniform across nations. However, significant gaps have been identified in the application of the extraterritoriality principle to protect HIC residents from being subjected to FGM overseas [33, 48].

The report by Leye and colleagues [37] includes an evaluation of a series information and training workshops for professionals from various sectors in five EU member countries to improve knowledge of the law as it relates to FGM. The evaluation suggests that building the capacities of various professionals involved in prevention, child protection, law enforcement and health such as social services, immigration officers, policemen, prosecutors, health providers and other professionals may lead to an increase in the number of prosecutions. In the EIGE study, outreach programs targeting parents, professionals and decision makers among FGM-practising communities were described. These activities involved education workshops and public forums that provided information about the penalties for perpetrators, the role of professionals in reporting of FGM and the role of extra-territoriality principle in the European Union, where courts can adjudicate on cases outside the territory of their country. Additionally, training of practitioners was delivered as part of the rollout of new guidelines on child protection laws. The study found that practitioners, and parents are becoming increasingly aware of the risk of prosecution for FGM [48]. The three studies discussing laws in this review suggest that legislation may work more effectively when viewed as a facilitator of protection against harmful practices and when used to conduct negotiations with the communities, health care workers and prosecutors.

### Interventions to support secondary prevention interventions

Five documents report on efforts to support safeguarding by improving citizen reporting of girls they suspect may be at risk of FGM to authorities. For example, Brown and Porter [34] in their report describe collaborative efforts in the UK that brought together, people from the voluntary and community sector, statutory agencies such as Safeguarding Boards, Metropolitan Police and health professionals, to strengthen community-based preventive work. These efforts comprise information sharing and the development of reporting or knowledge or suspicion of FGM risk to the authorities [34]. The NHS study reports on an audit undertaken of Clinical Commissioning Groups (CCG), one of which is the Croydon CCG services [43]. In the studies reviewed, the main focus of the interventions was on safeguarding that included FGM, building partnerships and the capacity of professionals involved (social welfare authorities, child protection officers, police officers, immigration services, school managers, legal professionals’ healthcare professionals), to improve protective mechanisms and engagement with communities and relevant social actors.

Training workshops and updates for professionals to strengthen mandatory reporting obligations to protect girls from FGM were described as an important adjunct to legislative actions. However, training for health, education and social workers and police across nations in the European Union was reported as haphazard and not conducted on a continuous, structured and nationwide basis. This is said to be hampered by the fact that in many Member States, health professionals cannot break their code of silence when the crime of FGM has already been performed, because FGM is not generally considered as a type of repetitive, recurrent child abuse [48].

McCracken and colleagues (2017) describe the development of pilot of FGM-specific assessment and intervention tools and protocols alongside training for relevant professionals. This was supported by community outreach activities to raise awareness of FGM-related issues, promote understanding of services and legislation. Health professionals provided talks at schools for pupils, parents, teachers and governors [39]. Other community-based efforts to facilitate these safeguarding measures include training FGM champions and creating safe spaces for girls and women at risk of FGM to talk about FGM. Bi-lingual community co-facilitators were engaged to communicate information about safeguarding in culturally appropriate ways and motivate community members to support the protection legislation and protocols [35].

### Interventions to support the tertiary prevention of FGM associated conditions

Only one report [39] included an evaluation of education provided to pregnant women and their partners who are eligible for de-infibulation to prevent obstetric complications. McCracken and colleagues (2017) describe the FGM Early Intervention Model delivered across three local authority areas in London. The authors found that dedicated FGM clinics within hospital midwifery services played a pivotal role in identifying women with FGM so that women could be provided with appropriate counselling and information about the law and available supportive services. During the pilot phase of the program, 235 women were seen in the FGM clinics. Practitioners were trained how to conduct risk assessment, identify individual women’s understandings of the practice and their education and information needs related to FGM and to report instances where children were believed to be at risk of FGM. Only immediate outcomes were reported, i.e. changes in health care professional knowledge and attitude, including their recognition of the need for sensitivity in direct work to avoid re-traumatising or alienating women who have undergone FGM, and other members of potentially-affected communities [39]. No information about changes in the knowledge and attitudes of women as a result of information provided by health care professionals is available.

#### Mapping interventions according to mode and focus

**Fig. 2:**
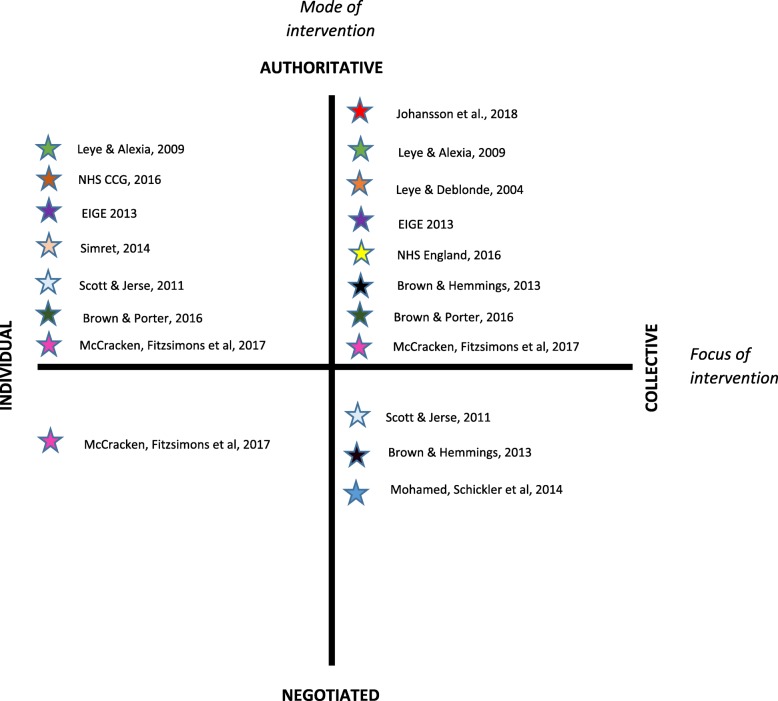
Mode and focus of the interventions evaluated in papers included in the review

Mapping the interventions described in the papers included in this review according to mode and foci of Beattie’s model [31], (see Fig. 2) provides insight into approaches taken to address FGM in HIC. The majority of the interventions (described in nine papers) occupy the top half of this framework and are authoritative in mode. In the top right quadrant, most focus on population level legal action, policy and reporting protocols for safeguarding girls to prevent FGM and prosecute those for procuring or intending to procure FGM. These include both primary and secondary prevention activities. Interventions described in six papers (in the top left quadrant) employ the authority of public-health expertise to re-direct the behaviour of community members and professionals in top-down prescriptive ways, such as the use of media to raise awareness, health education workshops and continuing professional development that are largely primary prevention strategies.

There are fewer interventions described in the reviewed papers that can be mapped to the negotiated lower half of the framework. Only one paper [39] describes individual personal counselling activities also noted previously as tertiary prevention directed at women who have FGM. Three papers outline interventions that focus on collective community development activities that are focused on primary prevention [35, 40, 41].

Several papers describe interventions with multiple modes and foci and are therefore represented in more than one quadrant. Three documents in the review [34, 37, 48] identified activities that are authoritative in mode with activities that focus on individual or groups and populations. Two documents [35, 41] describe authoritative, individually focused education workshops, as well as collective community development strategies with affected communities involving community workers. McCracken et al. [39] includes activities across all but one paradigm. Scott and Jerse [41] describe both individual and collective education interventions to address FGM in two quadrants while McCracken et al., describe personal counselling, professional training and safeguarding protocols that occupy three quadrants.

## Discussion

This review sought to gain insights into the characteristics of health interventions and associated outcomes. We examined programmatic approaches and strategies, target audiences and evaluation findings of interventions in HIC to improve the knowledge base to inform the design and evaluation of FGM health interventions. The majority of the reports included in this review described primary prevention activities in the UK and across Europe that are largely prescriptive or top-down forms of social intervention as compared with participative or ‘bottom-up’ forms. Emphasis is given to legislative action and strengthening the capacity of health care professionals, education and awareness raising for community members. This paradigm suggests the predominance of reformist and conservative ideologies and prescriptive structures. Beattie [31] depicts reformist legislative activities as “deprivation models” where bureaucratic interventions can address social injustice and work to protect those at risk of FGM. On the other hand, activities that favour health persuasion are focused on a “deficit model”, where selected individuals whose FGM knowledge and practices are regarded as lacking, are corrected. These approaches contrast with the few bottom-up and participative approaches identified in this review. Three papers [35, 40, 41] outline community development activities that support an “emancipation model” that mobilises community groups to design and deliver their own health promotion. One report identified tailored activities aimed at empowering individual women [39].

### Multifaceted comprehensive health promotion for FGM

While legislative action and health persuasion are important, the focus on these action paradigms do not appear to be justified by a strategic approach that is informed by baseline research. A focus on these paradigms may result in limited change and an unnecessary focus on risk factors that do not acknowledge the social determinants of health. Thomas and Stewart [49] have argued that a focus on these top-down approaches may lead to “victim blaming” and can work to disempower those most affected, in this case migrant and refugee women and girls from countries where FGM is practiced. FGM is a complex issue that requires a multifaceted response that encompasses socio-economic, physical and environmental factors, education and learning, services and facilities, and community activities. Beattie’s model supports this concept and illustrates that approaches across all quadrants and axes are required for holistic, well-rounded health promotion policy and practice. A health promotion approach is therefore required that considers prevention activities as part of a holistic program of action to prevent FGM but also engage communities in positive health enhancement.

### Evidence to prevent FGM

This review was not able to establish the effectiveness of one intervention over another, or the cumulative effects of one or several interventions on the prevalence of FGM. The evidence on the interventions outlined in the reports included in this review is therefore limited. The assessments are largely process evaluations that include measures of short-term outcomes or audits of practices. Much of the available evidence has focused on measuring immediate outputs such as pre and post intervention changes in attitude, knowledge and confidence. It points to the possibility of these interventions being useful in helping migrant communities abandon this harmful practice. Reports examining FGM legislation are largely descriptive overviews of the current legal and policy situation in HIC that has recently been captured in the World Bank’s Compendium on international and national legal frameworks on FGM and in a recent text [50, 51]. These evaluations have been hampered by a lack of monitoring and reporting systems [33] and the implementation of legislation, affected by a lack of clear guidelines, legal ambiguity, and reluctance to report parents and community members. A review by Balfour and others also shows that the evidence on educational/promotional interventions is weak and mainly focuses on surrogate markers of opinion change etc. [52]. This review included only peer reviewed evidence and as such included only two studies. This review has shown little insight into the long-term impact of single or multiple health prevention activities. Given the significant amount of effort and resources spent on programs specifically designed to promote the abandonment of FGM, there is need to strengthen research designs to include more robust methodologies, community driven, cross-disciplinary and international collaborative studies which take into consideration not just the immediate outcomes but the impact of the interventions.

### Involving women and girls in the design and evaluation of health prevention

Our review located one report [39] that examined outcomes of an initiative that involved the training of health professionals to provide education and information during consultations with pregnant women who have experienced FGM. While this sheds some light on the importance of improving the skills of professionals to provide quality care as emphasised in WHO guidelines [1], evidence is needed concerning the experience of women and girls and their values and preferences during clinical consultations. A recent review has examined the maternity care experiences and health needs of women with FGM in HIC [53]. Another study on health information and education interventions for FGM included women’s perspectives [54]. While the papers included in these reviews do not investigate the outcomes of specific interventions, rather current care, they do indicate that there is much to be done to better meet women’s needs for health promotion. Both reviews highlight poor provider communication, negative attitudes and a lack of knowledge about FGM. If women centred, culturally appropriate health promotion including prevention is to be delivered during consultations that empowers women to take control over their own health, then women’s needs must be considered in such interactions. Women should be involved in the design and evaluation of education and information resources [55].

### Multi-agency response to FGM

The majority of the interventions in our review involve a wide range of professionals from multiple agencies, including health professionals, police, social workers and school teachers across the public and non-state sector. This cross-sector approach to FGM prevention was highlighted in the evaluations of safeguarding practices from the UK where collaboration was fostered through networking, linking national child protection policy with organization policy, and training initiatives. However, central to many of these efforts were activities to foster engagement with the local community through outreach and the involvement of community champions and advocates to raise awareness of safeguarding laws and reporting processes.

There are a number of documents to guideline multiagency response in HIC. In the UK, the Government has produced Multi-agency Statutory Guidelines for frontline professionals [56] working to safeguard children at risk from FGM in England and Wales. In Spain, an Action protocol for the prevention of FGM [57] lays out ways in which health care, education, social services, child care services and security professionals can collaborate to identify and report children at risk in Catalunya. Health professional themselves have also produced guidelines for inter-professional collaboration for safeguarding such as the Intercollegiate recommendations for identifying, recording and reporting in the UK [58]. In Australia, service coordination emphasises guidelines have been produced to facilitated collaborative partnerships between services and organisations in the community [59].

### Best practice health prevention guidelines for FGM

The interventions in this review were largely guided by the law as it relates to FGM and clinical practice guidelines. However, no mention is made of guidelines in this area. In Australia, the National *Education Toolkit for FGM/C Awareness (NETFA) Best Practice Guide* provides nationally accepted benchmarks for culturally appropriate FGM prevention programs for community organisations [60]. These ten standards include the need for resources to be inclusive and interactive, relevant, accurate, respectful of human rights and cultural dignity and empowering. The European commission through the REPLACE Approach provides a toolkit or ‘how to’ guide for community members affected by FGM, and community leaders and organisations working with them to bring about an end to FGM in the EU. [18]. The REPLACE approach has three pillars that emphasise behavioural change that recognises the socio-cultural context and social norm transformation, the importance of engaging and working with communities and the need for evaluation to inform all stages of programming.

### Limitations

One of the limitations of our study is paucity of peer reviewed evidence for the research topic. The majority of the evidence retrieved for this study is therefore from grey literature, specifically from programmatic evaluations undertaken by consultants. As such, quality assessment may be variable, affecting the analysis. However, two authors appraised the studies independently to ensure the quality of reporting, methodological, rigour and conceptual depth and breadth of all included studies. Secondly, given the heterogeneous nature of the retrieved evidence (methods, purposes, between programs, target audiences, duration and content), it was difficult to make firm conclusions, particularly as many of the outcomes and measures for determining effectiveness varied widely. However, the inclusion of individual case studies of programs, provides rich, descriptive evidence of interventions, a useful contribution to address the questions on how governments, public health entities and providers have attempted to address the FGM in HIC.

## Conclusion

Studies show that current evaluations conducted in HIC are mainly of primary preventions and top-down approaches that seek to employ the authority of public health expertise to re-direct the behaviour of community members and professionals. Few interventions focused on collective activities within communities or were aimed at empowering individual women. To streamline all the efforts in practice, there is a clear need for an integrated health promotion approach to best organise the multifaceted, multilayered and often overlapping public health interventions to address the medical, social and cultural issues involved in curtailing FGM. Moreover, evidence of the effectiveness of interventions on reducing the prevalence of FGM is lacking highlighting the need for investment in impact evaluation and rigorous study designs.

Beattie’s framework may provide a useful way of guiding and articulating negotiated and authoritative health promotion actions required to address FGM at national and local levels. Such a framework would promote partnerships, synergies and communication between all stakeholders including education, health, police, social care agencies and the community to improve awareness and support for women and girls. Co-ordination would also promote greater efficiency in the implementation of interventions and avoid duplication of efforts. In addition, a comprehensive approach could potentially improve protocols, guidelines and learning materials, foster consistent and rigorous data collection and reporting on FGM, including information about girls at risk of, or girls who have already undergone FGM. This information would equip frontline service and healthcare professionals to effectively protect and care for girls and women in regard to FGM, intervene and effectively meet mandatory reporting requirements. Such a framework would bring logic, clarity, comprehension, evidence and an economically more effective response for current and future health promotion interventions addressing FGM in HIC.

## Data Availability

Data generated and analysed during the study are included in this manuscript and its supplementary information files.

## Abbreviations

AACODS: Accuracy, Coverage, Objectivity, Date, and Significance
CASP: Critical Appraisal Skills Programme
EBSCO: Elton B. Stephens Co.
FGM/FGC: Female Genital Mutilation /Cutting
HIC: High Income Country
PubMED: MEDLINE database of references and abstracts on life sciences and biomedical
